# Supplementary material for: Collective skyrmion motion under the influence of an additional interfacial spin-transfer torque
Source: Sci Rep. 2022 Jun 24;12:10786. doi: 10.1038/s41598-022-14969-2 (PMC9232533; doi:10.1038/s41598-022-14969-2)
Supplement: Supplementary file 1 — Supplementary Information. [file 41598_2022_14969_MOESM1_ESM.docx]

Supplementary Information

Collective skyrmion motion under the influence of an additional interfacial spin-transfer torque

Callum R. MacKinnon^1,*^, Katharina Zeissler^2,3^, Simone Finizio^4^, Jörg Raabe^4^, Christopher H. Marrows^2,3^, Tim Mercer^1^, Philip R. Bissell^1^ & Serban Lepadatu^1,**^

^1^ *Jeremiah Horrocks Institute for Mathematics, Physics and Astronomy, University of Central Lancashire, Preston PR1 2HE, U.K.*

^2^ *School of Physics and Astronomy, University of Leeds, Leeds LS2 9JT, UK*

*^3^Bragg Center for Materials Research, University of Leeds, Leeds LS2 9JT, UK*

*^4^Swiss Light Source, Paul Scherrer Institut, 5232 Villigen, Switzerland*

Contributions of SOT, ISTT, and STT

The effects of different spin torques acting on a single skyrmion, in a Pt(2.7 nm)/Co(d_Co_)/Ir(0.4 nm) multilayer with d_Co_ ranging from 5 Å to 13 Å, are shown in Fig. S1 for a skyrmion with 100 nm diameter. The different spin torques are computed self-consistently using the spin transport solver for each value of d_Co_, and skyrmion velocities and SkHA at *J_C_* = 2.5×10^11^ A/m^2^ are shown in Fig. S1 as polar plots, for the individual spin torques and combinations. As expected, the paths under the combined spin torques are exactly those obtained by vector addition of the paths taken under the individual spin torques. The effect of the bulk STT is independent of Co layer thickness, and significantly smaller than those of SOT and ISTT. However, SOT and ISTT are strongly dependent on the Co layer thickness, and rapidly decrease in strength with increasing Co layer thickness, as expected from the interfacial spin torque in Equation **(**1**)**:

| $\mathbf{T}_{S}=\frac{g\mu_{B}}{ed_{F}}\left[\mathrm{Re}\left\{G^{\uparrow\downarrow}\right\}\mathbf{m}\times\left(\mathbf{m}\times\Delta\mathbf{V}_{S}\right)+\mathrm{Im}\left\{G^{\uparrow\downarrow}\right\}\mathbf{m}\times\Delta\mathbf{V}_{S}\right]$. | **(**1**)** |
| --- | --- |

For d_Co_ = 8 Å, as used for the results in the main text, both SOT and ISTT are significantly stronger than bulk STT, and it may be seen from Fig. S1(b) how both the SkHA and skyrmion velocity are affected for the full spin torque model, when compared to the SOT-only model.


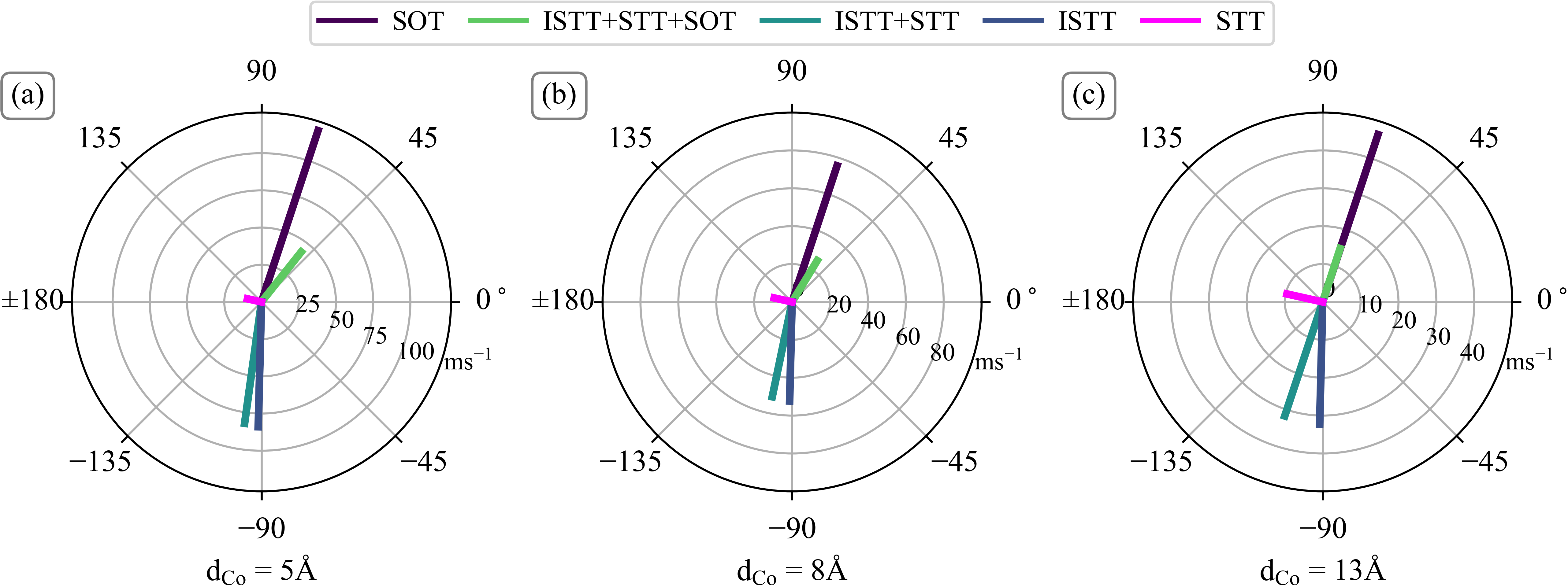


**Figure S1**. Effect of individual torques on a 100 nm diameter skyrmion, and combined effect, for Co layer of different thickness, (a) 5 Å, (b) 8 Å, and (c) 13 Å. A damping value of *α* = 0.1 was used here, with a driving current density of *J_C_* = 2.5×10^11^ A/m^2^. The polar plots show the skyrmion velocity, obtained using the Thiele model.

The effect of the different spin torques is also strongly dependent on the skyrmion diameter. SkHA and skyrmion velocity values are shown in Fig. S2 as a function of diameter, obtained using the Thiele model of Equation **(**2**)**:

| $v=\sqrt{\frac{\left(u_{SOT}-\beta_{STT}Du_{STT}\right)^{2}+u_{STT}^{2}}{1+\left(\alpha D\right)^{2}}}$,  $\tan\left(\theta_{SkHA}\right)=\frac{u_{SOT}+\left(\alpha-\beta_{STT}\right)Du_{STT}}{\alpha Du_{SOT}-(1+\alpha\beta_{STT}D^{2})u_{STT}}$. | **(**2**)** |
| --- | --- |

As expected, the SOT-only model shows a monotonic increase in SkHA with decreasing diameter. On the other hand, the full spin torque model shows a much flatter SkHA-diameter dependence, and for diameters below 100 nm shows a decrease in SkHA with decreasing diameter. Moreover, skyrmion velocities under the full spin torque are much smaller than those of the SOT-only model, and as discussed in the main text more closely aligns with experimental results, particularly when the effect of skyrmion collections on group velocity is taken into account.


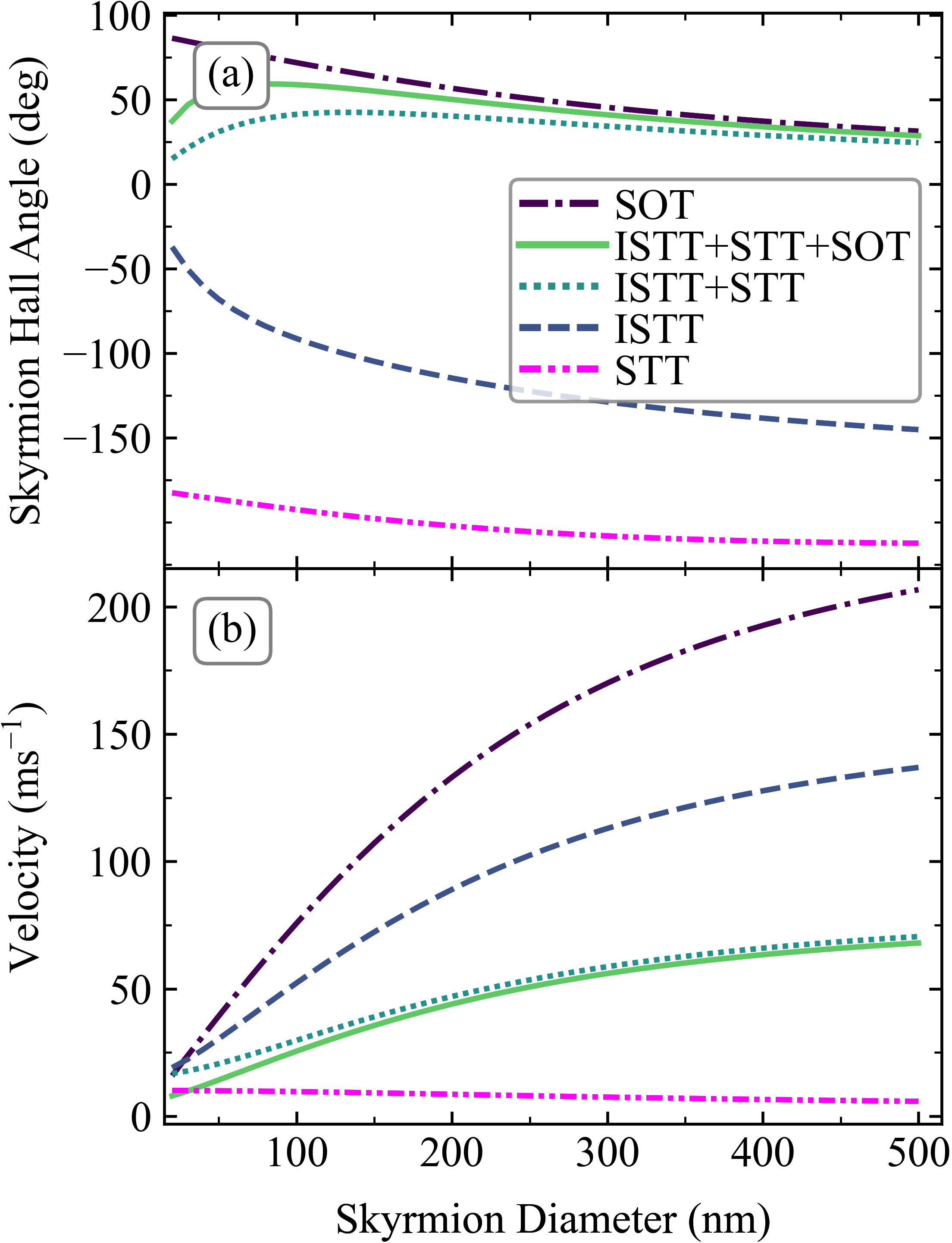


**Figure S2**. Variation of (a) SkHA, and (b) skyrmion velocity, with skyrmion diameter for the individual spin torques and combinations, obtained using the Thiele model for Co layer thickness of 8 Å. A damping value of *α* = 0.1 was used here, with a driving current density of *J_C_* = 2.5×10^11^ A/m^2^. SkHA and velocity as a function of diameter are obtained using the Thiele model.

We also note that isolated skyrmion velocities obtained in other experiments align closely with the full spin torque model, whilst the SOT-only model predicts much larger velocities at damping values of *α* = 0.1 and lower, although disorder also plays a part in reducing velocities. For example Ref. [[[1]](#endnote-2)] shows ~40 ms-1 at *J_C_* = 2.5×10^11^ A/m^2^ with skyrmion diameters 100-150 nm, Ref. [[[2]](#endnote-3)] shows < 30 ms-1 at *J_C_* = 5×10^11^ A/m^2^ and room temperature, with skyrmion diameters 100-150 nm, Ref. [[[3]](#endnote-4)] shows ~30 ms-1 at *J_C_* = 5×10^11^ A/m^2^ with skyrmion diameters 100-150 nm, and Ref. [[[4]](#endnote-5)] with stacks containing Pt(3 nm)/Co(0.9 nm)/Ta(4 nm), and also for Pt(4.5 nm)/CoFeB(0.7 nm)/MgO(1.4 nm), shows ~20 ms^-1^ at *J_C_* = 2.5×10^11^ A/m^2^ with skyrmion diameters 100-150 nm.

Finally, we show the effect of damping on the SkHA in Fig. S3. As discussed in the main text, increasing damping results in smaller SkHA values as expected, both for the SOT-only as well as the full spin torque models. However, even for large values of *α* = 0.3, the SOT-only model cannot explain the small SkHA values obtained for small diameter skyrmions, showing instead a rapidly increasing SkHA as the diameter decreases. As discussed in the main text, the full spin torque reproduces experimental results with a realistic damping value (*α* = 0.1) when comparing modelling results in the elastic depinning regime to experimental results at the same current density, whilst the SOT-only model results in ~50° discrepancy.


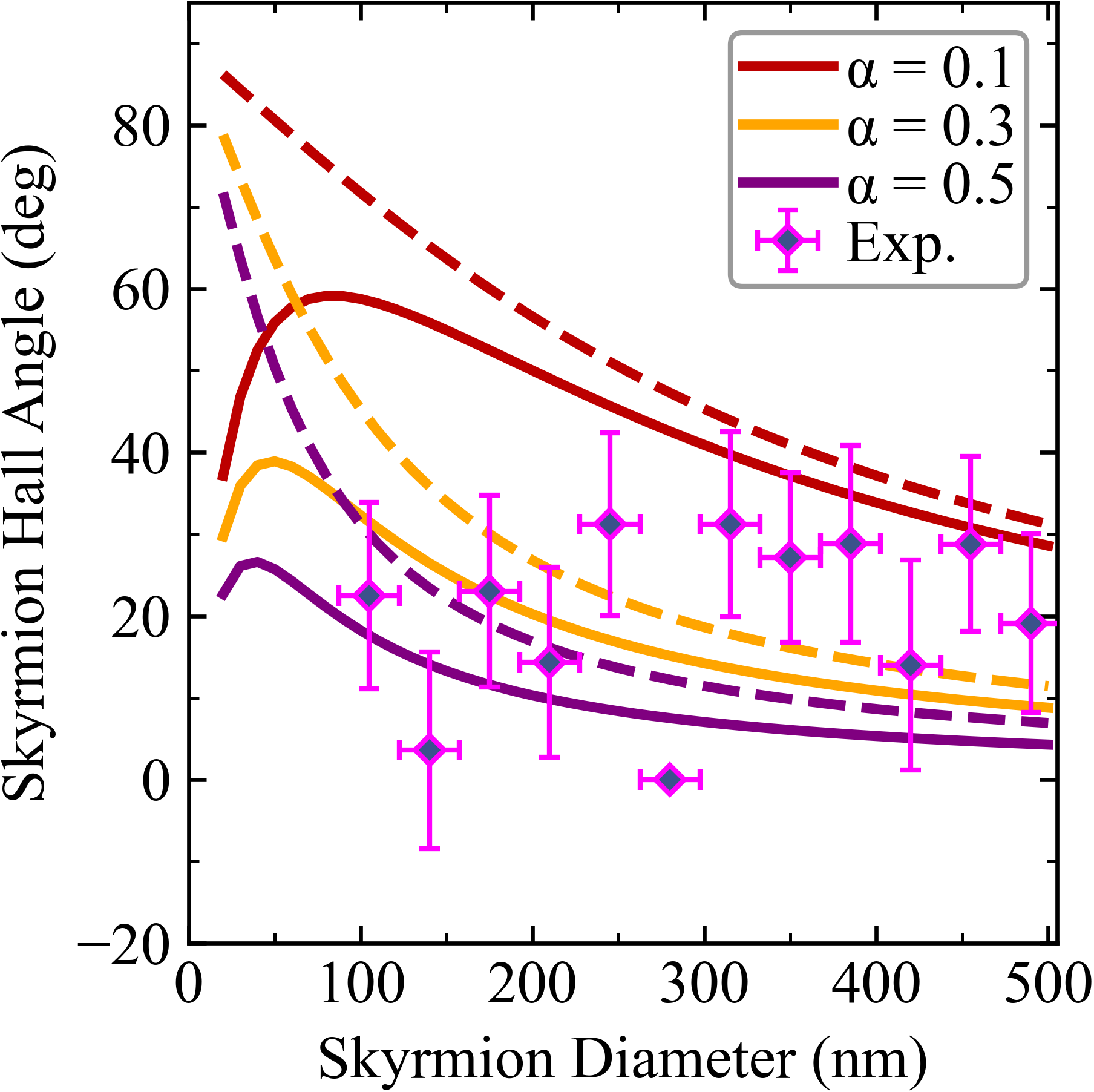


**Figure S3**. Variation of SkHA as a function of skyrmion diameter for FM layer thickness of 8 Å, where the dashed lines are the SOT-only contribution, whilst the solid lines are the full SOT+ISTT+STT results. The damping value is varied from 0.1 up to 0.5, with Thiele model results compared with the experimental data.

Effect of transport parameters on SkHA and velocity

The material parameters used to model spin transport in the Pt(2.7 nm)/Co(*d_Co_*)/Ir(0.4 nm) multilayer are shown in Table S1. In order to investigate the effect of various material parameters and layer thicknesses on the SkHA and skyrmion velocities, we concentrate on the most important parameters which have a marked effect on skyrmion motion: Pt and Co layer thicknesses, Pt spin flip length, and spin mixing conductance at the Co/Pt interface; the effect of magnetic damping has already been investigated in Appendix A. The contribution from the Ir layer (and Ir/Co interface) is small compared to that from Pt.

**Table S1**. Transport parameters used to model spin transport in the Pt/Co/Ir stack.

| **Transport Parameters (Co)** | **Value** |
| --- | --- |
| *σ* | 5 M S/m [[[5]](#endnote-6)] |
| *D_e_* | 0.001 m^2^/s [[[6]](#endnote-7)] |
| *λ_sf_* | 42 nm [5] |
| *λ_J_* | 2 nm [[[7]](#endnote-8)] |
| *λ_φ_* | 3.2 nm [[[8]](#endnote-9)] |
| **Transport Parameters (Pt)** |  |
| *σ* | 7 MS/m [[[9]](#endnote-10)] |
| *D_e_* | 0.004 m^2^/s [6] |
| *λ_sf_* | 1.4 nm [9] |
| *θ_SHA_* | 0.19 [9] |
| *G*^↑↓^ (Pt/Co) | 1.5 + *i*0.45 PS/m^2^ [9,3,[[10]](#endnote-11)] |
| **Transport Parameters (Ir)** |  |
| *σ* | 1.4 MS/m [[[11]](#endnote-12)] |
| *D_e_* | 0.0002 m^2^/s [6] |
| *λ_sf_* | 0.5 nm [[[12]](#endnote-13)] |
| *θ_SHA_* | 0.02 [12] |
| *G*^↑↓^ (Co/Ir) | 0.35 + *i*0.045 PS/m^2^ [[[13]](#endnote-14)] |

Here, a 100 nm diameter skyrmion is set, and for each combination of material parameters the spin transport solver is used to compute the various spin torques self-consistently using the equations discussed in the main text. The SOT, STT, and ISTT are then fitted to the computed spin torques in order to extract their respective spin torque parameters. Using the spin torque parameters, the Thiele model – Equation (2) – is then used to compute the SkHA and skyrmion velocities. Results are shown in Figs. S4 – S7. It must be emphasized, the results shown below are obtained for ideal and isolated skyrmions, without landscape disorder, and are thus limiting cases. However, this analysis is useful for understanding the effect of the different spin torques and combinations, for experimentally relevant material parameters and associated uncertainty ranges.


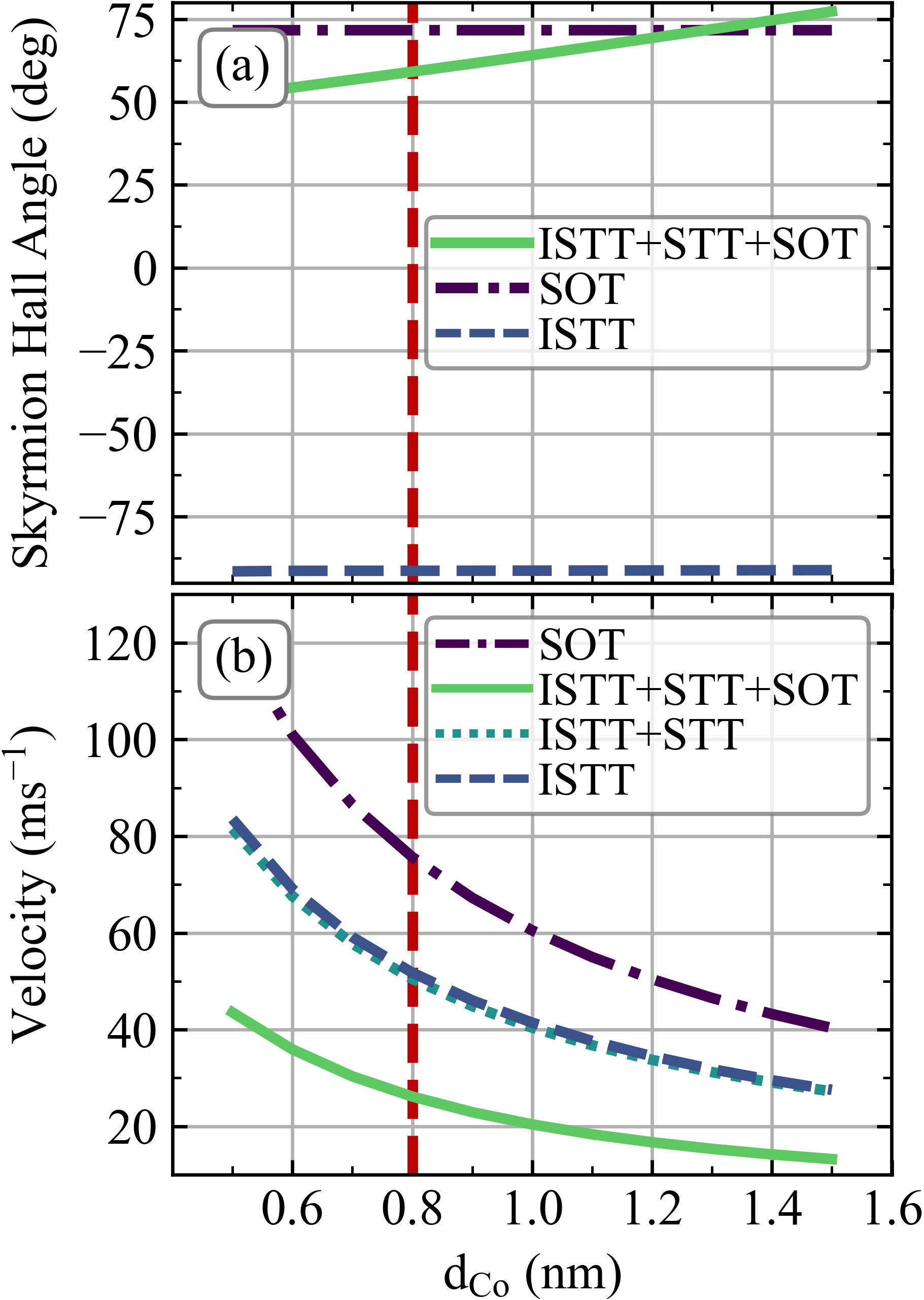


**Figure S4**. Effect of Co layer thickness, d_Co_, on (a) SkHA, and (b) velocity (at *J_C_* = 2.5×10^11^ A/m^2^) for various spin torques and combinations. The dashed vertical line shows the value used for the results in the main text.

Varying the Co layer thickness results in an inverse dependence of the spin torque strengths, as expected from Equation (1), which leads to an inverse dependence of the skyrmion velocity – Fig. S4(b). By comparing the velocities obtained with ISTT+STT and ISTT-only, we can see the effect of the bulk STT on skyrmion velocities is relatively negligible. For SOT-only and ISTT-only, the SkHA value is constant – Fig. S4(a) – however, when the full spin torque is considered, a linear dependence on the Co layer thickness results. This arises since the motion under the combined spin torques is obtained as vector addition of the motion under the separate spin torques; thus, a variation in their relative velocities results in a variation of the SkHA under the combined spin torques.


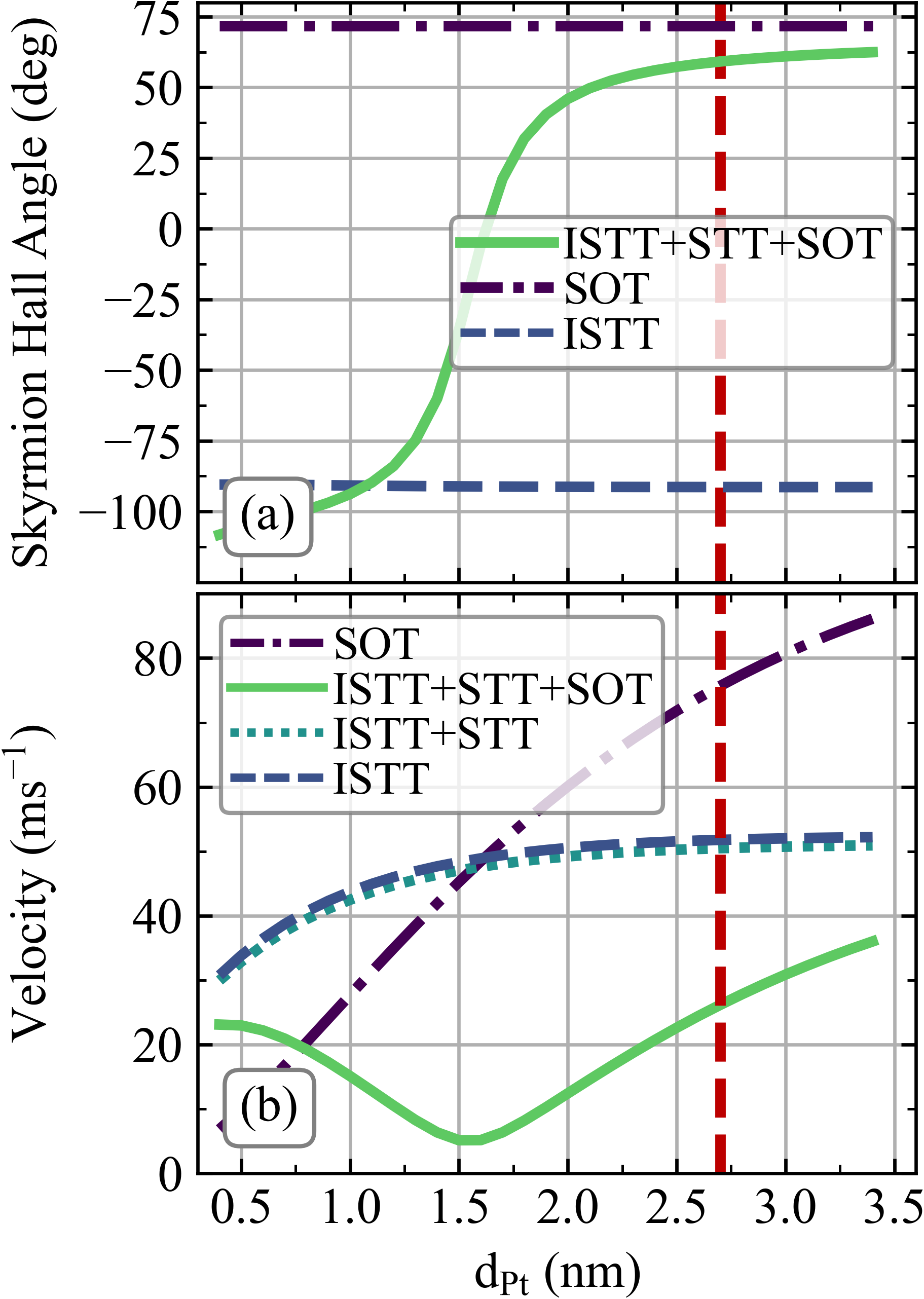


**Figure S5**. Effect of Pt layer thickness, d_Pt_, on (a) SkHA, and (b) velocity (at *J_C_* = 2.5×10^11^ A/m^2^) for various spin torques and combinations. The dashed vertical line shows the value used for the results in the main text.

Varying the Pt layer thickness results in a change in the SOT and ISTT strength, primarily due to a change in the spin accumulation at the Pt/Co interface, which is strongly dependent on the spin-flip length to layer thickness ratio. For the SOT this results in a change in the effective SHA, *θ_SHAeff_*, obtained by solving the drift-diffusion model as [10]:

| . | **(**3**)** |
| --- | --- |

Here $N_{\lambda}=\tanh\left(d_{Pt}/\lambda_{sf,Pt}\right)/\lambda_{sf,Pt}$ and $\tilde{G}=2G^{\uparrow\downarrow}/\sigma_{Pt}$. Thus, for example, with an intrinsic Pt SHA value of *θ_SHA_* = 0.19, *θ_SHAeff_* = 0.048 at 2.7 nm thickness, but this decreases to *θ_SHAeff_* = 0.006 at 0.5 nm thickness. This results in a monotonic dependence of the skyrmion velocity with Pt thickness under SOT, and similarly for ISTT – Fig. S5(b). As the Pt layer thickness is decreased, eventually the velocity contribution from ISTT becomes dominant (noting that an ISTT contribution from the 0.4 nm thick Ir layer is also present). This explains the marked change in SkHA seen in Fig. S5(a). These results show it is possible to control the SkHA by varying the relative strengths of the contributing spin torques. However, zero SkHA desired for applications occurs close to the point when the effects of SOT and ISTT nearly cancel out, and is thus accompanied by very low skyrmion velocities, as seen in Fig. S5.


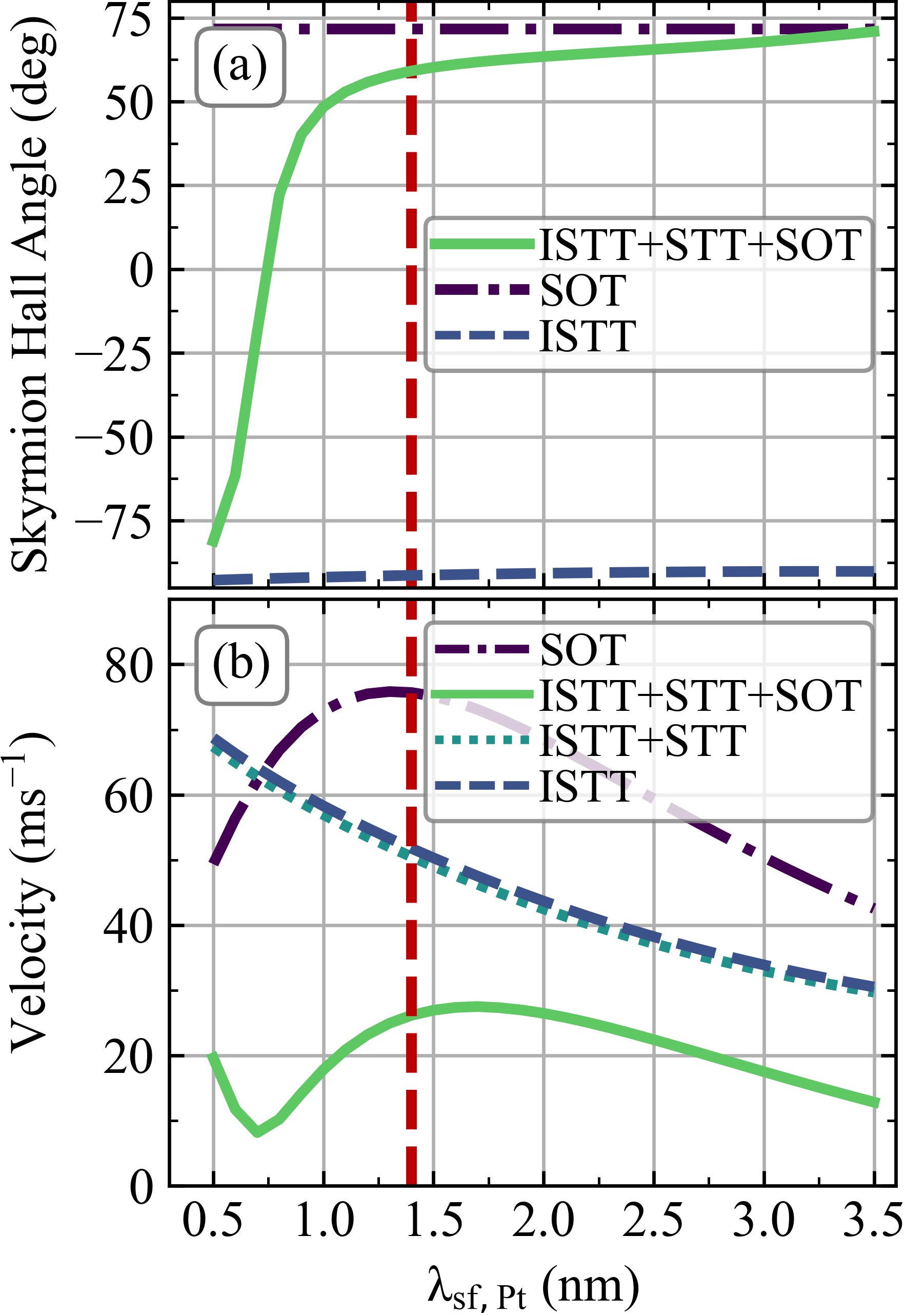


**Figure S6**. Effect of Pt spin flip length, *λ_sf,Pt_*, on (a) SkHA, and (b) velocity (at *J_C_* = 2.5×10^11^ A/m^2^) for various spin torques and combinations. The dashed vertical line shows the value used for the results in the main text.

Similar remarks also apply to the dependence on Pt spin-flip length, shown in Fig. S6. The ISTT depends inversely on the spin-flip length, since smaller values result in larger spin accumulation gradients at the interface, and thus stronger diffusive vertical spin currents. The SOT strength also depends on the spin-flip length, as given by Equation **(**3), noting that *θ_SHAeff_* depends not just on the *d_Pt_* / *λ_sf,Pt_* ratio, but also on *λ_sf,Pt_* separately.


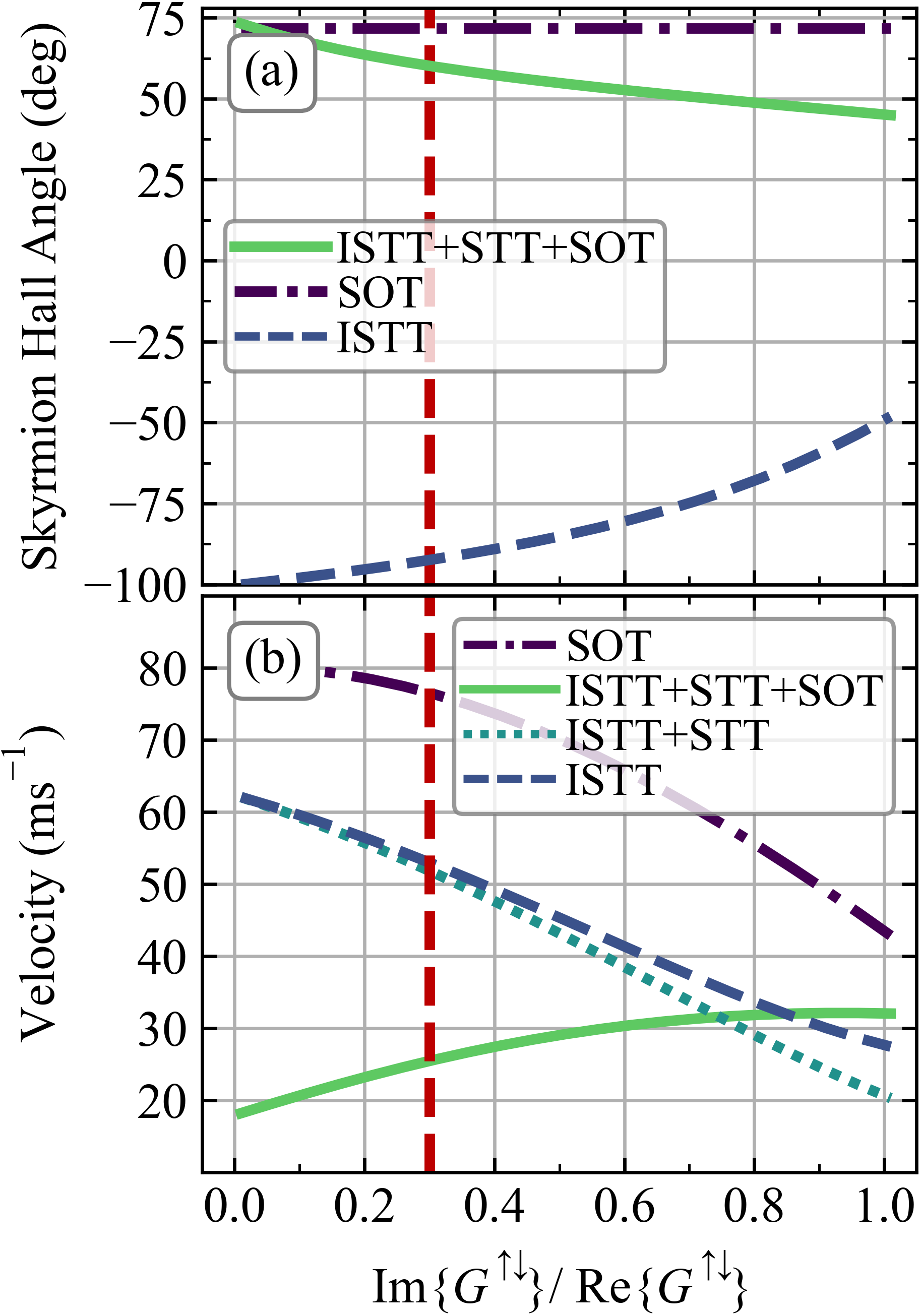


**Figure S7**. Effect of spin mixing conductance imaginary to real part ratio on (a) SkHA, and (b) velocity (at *J_C_* = 2.5×10^11^ A/m^2^) for various spin torques and combinations. The dashed vertical line shows the value used for the results in the main text.

The spin mixing conductance, *G*^↑↓^, also has an important effect on interfacial spin torques, Equation (1). Experimental data on the real part of *G*^↑↓^ are readily available in the literature, e.g. Ref. [9] for Pt/Co interfaces. On the other hand, data on the imaginary part of *G*^↑↓^ are scarcer. In this work we obtain Im{*G*^↑↓^} by using experimental data on the FL-SOT to DL-SOT ratio, e.g. Ref. [3]. The FL-SOT coefficient, *r_G_*, may be obtained by solving the drift-diffusion model as:

| . | **(**4**)** |
| --- | --- |

From experimental values of *r_G_*, this can be solved to obtain Im{*G*^↑↓^}. The effect of the imaginary part of the spin-mixing conductance is shown in Fig. S7, by plotting the SkHA and velocity as a function of the ratio *r* = Im{*G*^↑↓^} / Re{*G*^↑↓^}. Decreasing *r* results in stronger SOT and ISTT. At the same time, the interfacial non-adiabaticity parameter, *β*_⊥_, also depends on *r* [[[14]](#endnote-15)], which results in a change of the SkHA under ISTT-only, unlike for SOT-only. For very small values of *r* the effect of SOT and ISTT are nearly opposite, which results in skyrmion velocities tending towards zero under the combined spin torque.

Effect of Stack Repetition on Skyrmions and Transport Parameters

It is known that stacks with multiple ferromagnetic layer repetitions can result in a hybrid skyrmion, where the chirality changes between bottom and top layers [[[15]](#endnote-16)]. With the DMI constant of -1.1 mJ/m^2^, a skyrmion is stabilized in a stack with 8 Co layers, each 0.8 nm thick, and with 1 nm spacing, as for the measured stack. The dipolar field between the layers was computed exactly using the multi-layered convolution algorithm previously developed [[[16]](#endnote-17)]. The magnetization profile through the center of the skyrmion is plotted in Fig. S8, showing the *m_x_* component for different layers. These results confirm the homochiral Néel skyrmion structure expected for samples with a high DMI constant.

**Figure S8**. Normalized *x* component of magnetization profile through the centre of the skyrmion along the same direction. A stack with 8 repetitions of Co layer is used here, and the profile is plotted for different layers (numbered 1 through 8).


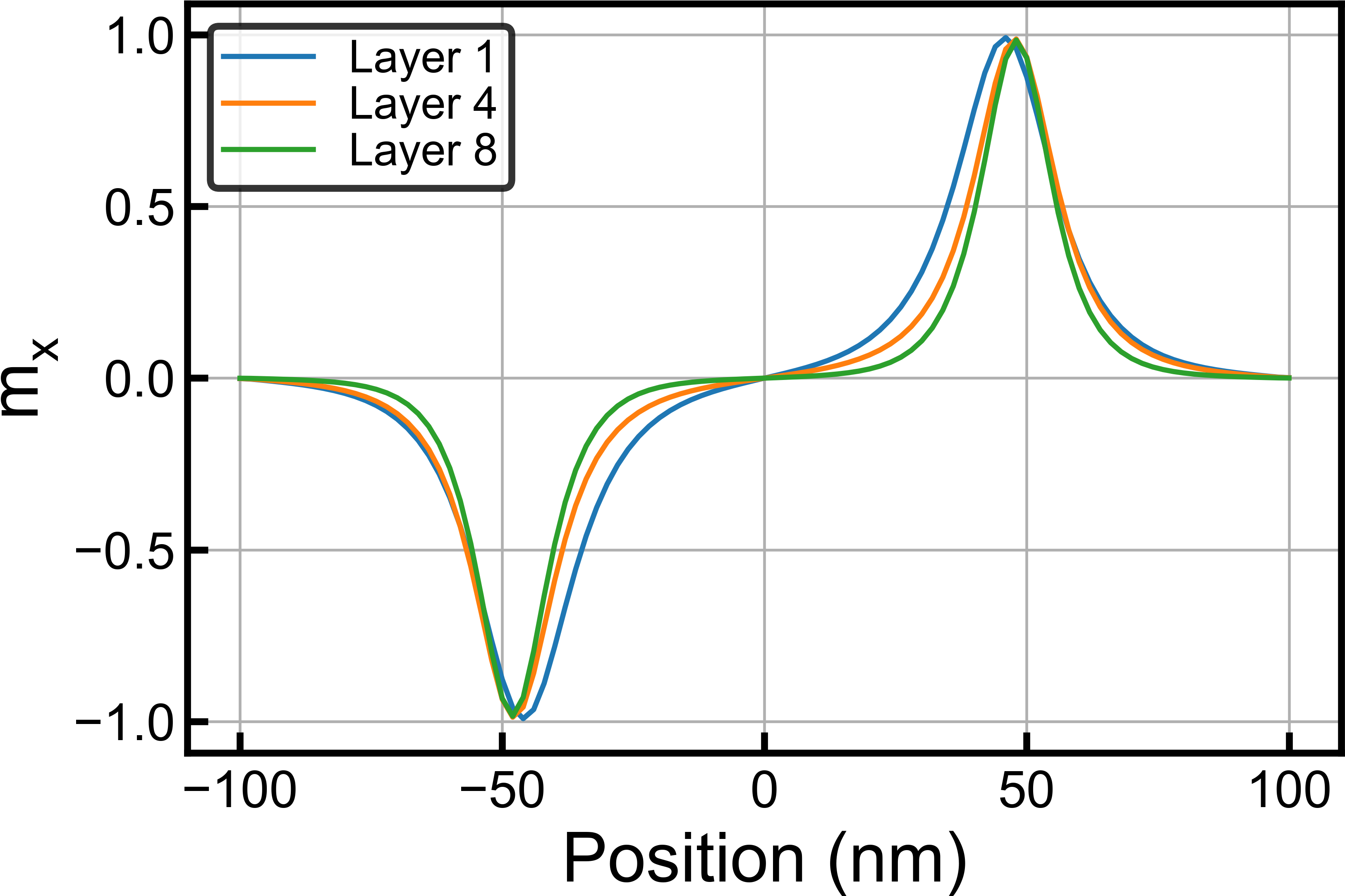


As the number of stack repetitions increases, another expected effect is an increase in the skyrmion diameter for the same external field, due to the decrease in the effective anisotropy [4]. When a skyrmion collection is considered however, this effect is not as pronounced, due to a confinement effect from repulsive interactions with neighbouring skyrmions. To analyse this, we calculate the average skyrmion diameter, as well as spread, for skyrmion collections as a function of number of stack repetitions in a Pt(2.7)/[Co(0.8)/Ir(0.4)/Pt(0.6)]_×n_/Pt(2.2) stack. Results are shown in Fig. S9. As may be seen, the average skyrmion diameter increases as the number of stack repetitions is increased, reaching a constant value after 5 repetitions. This effect is relatively small however, with an increase in the average skyrmion diameter of ~50 nm. As was noted in the main text, the experimental samples have on average larger skyrmions – see Fig. 2(c) in the main text – and inclusion of a stack repetition would more closely align the experiment and modelling data.

**Figure S9**. Average skyrmion diameter computed for skyrmion collections with densities *N* = 12.5 Sk/μm^2^ and 25 Sk/μm^2^ respectively, and out-of-plane field of *H* = 1.2 kA/m, as a function of number of stack repetitions in Pt(2.7)/[Co(0.8)/Ir(0.4)/Pt(0.6)]_×n_/Pt(2.2). The error bars indicate the quartile 1 to quartile 3 spread.


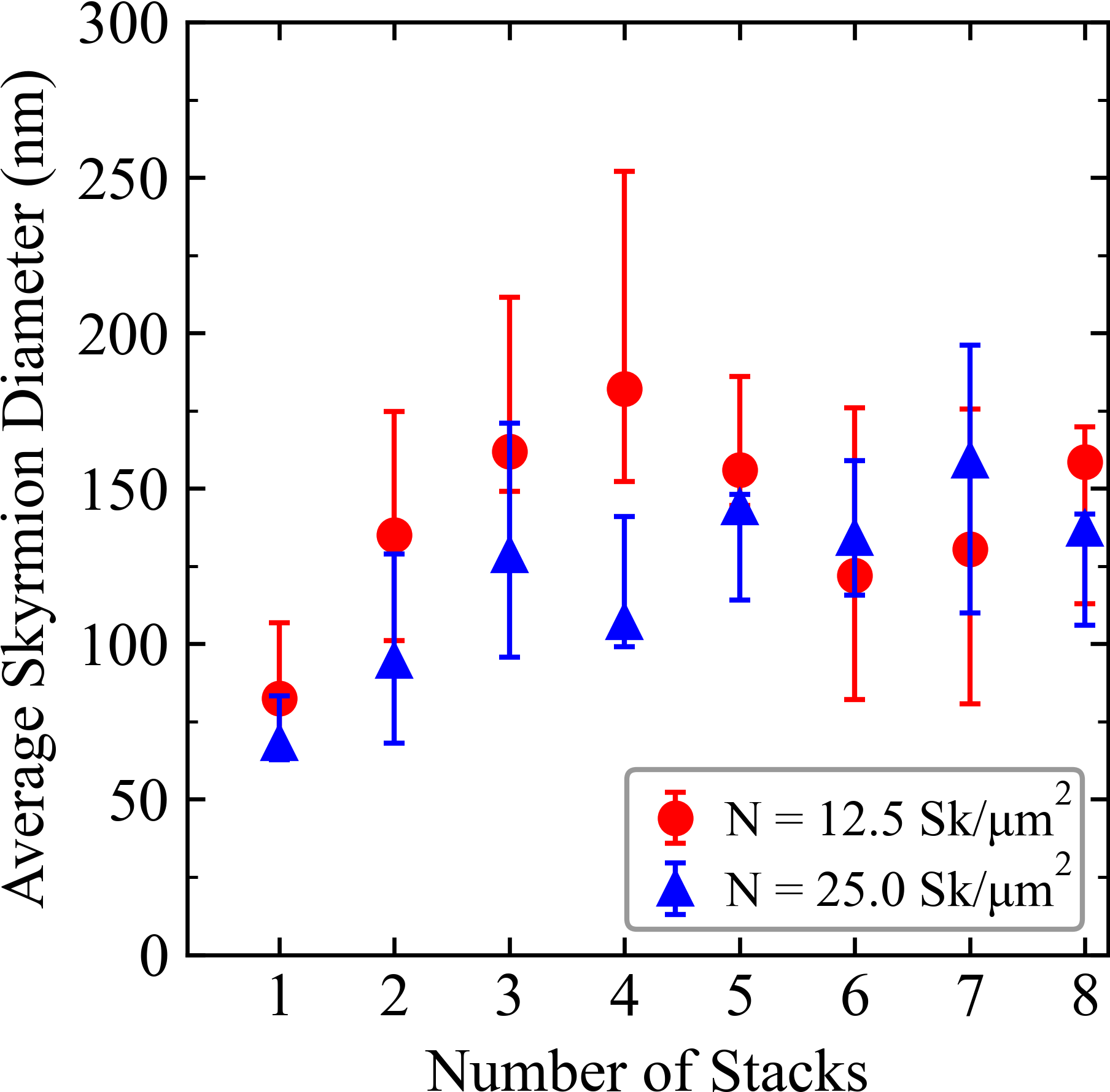


Finally, the effect on spin torques from stack repetition is discussed. With a single stack repetition, the spin torque parameters extracted by fitting the self-consistent computed spin torque, are *θ_SHAeff_* = 0.048, *r_G_* = 0.45, *P_ISTT_* = -1.34, *β_ISTT_* = -0.96 (effective ISTT polarization $\tilde{P}_{ISTT}=P_{ISTT}/(1+\beta_{ISTT}^{2})=-0.69$). When a stack repetition is introduced, e.g. for 8 repetitions, the new parameters are *θ_SHAeff_* = 0.046, *r_G_* = 0.47, *P_ISTT_* = -1.25, *β_ISTT_* = -0.95 ($\tilde{P}_{ISTT}=-0.66$). The bulk STT values are *P* = 0.42 and *β =* 0.002 ($\tilde{P}=0.42$) in all cases.

A very small decrease in the effective spin Hall angle is obtained, since the value is largely set by the thicker Pt underlayer (2.7 nm thickness). The effect of the thinner Pt layer (0.6 nm) from the next repetition, is to reduce the SOT strength, however this effect is negligible. With ISTT, vertical spin currents generated at skyrmions in one Co layer, traverse the adjacent Ir and Pt layers, reaching the neighbouring Co layer from the next stack repetition. The effect on the total ISTT is small however, as may be seen by comparing the extracted spin torque parameters above, for two reasons. i) The spin-flip lengths of Pt and Ir are small, which results in a rapid exponential decay of spin accumulation. Thus, whilst the diffusive spin currents originating in the Co layer are large close to the interface, they are smaller when reaching the neighbouring Co layer. ii) Transverse components of spin accumulation are absorbed at the interface, resulting in ISTT, and thus these diffusive spin currents in Ir and Pt are largely of longitudinal polarization. Since the Co layers have to a very good approximation the same magnetization texture, a negligible additional contribution to ISTT results.

STXM Imaging of Current-Induced Skyrmion Movement

An example of the experimental skyrmion path measurement is shown in Fig. S10. After nucleation of skyrmions, an initial state is imaged, as shown in Fig. S10(b), with analysed skyrmions identified using circles. Then, the motion image sequence was taken by applying three 22-ns-long current pulses, separated by a delay of 2 μs, with a measured pulse shown in Fig. S10(a). Static single helicity STXM images of the magnetization state of the wire were taken after each sequence. This sequence was then repeated 19 times, and analysis of the generated images using the TrackMate algorithm, allows extraction of skyrmion paths. These are shown in Fig. S10(b), where each path is plotted using different colors.

**Figure S10**. Experimental measurement of skyrmion movement. (a) Current pulse used to drive skyrmions, (b) STXM image of skyrmions, identified using circles, with skyrmion paths identified using different colors, extracted using the TrackMate algorithm from multiple STXM images. An out-of-plane field of *H* = 1.2 kA/m was used here.


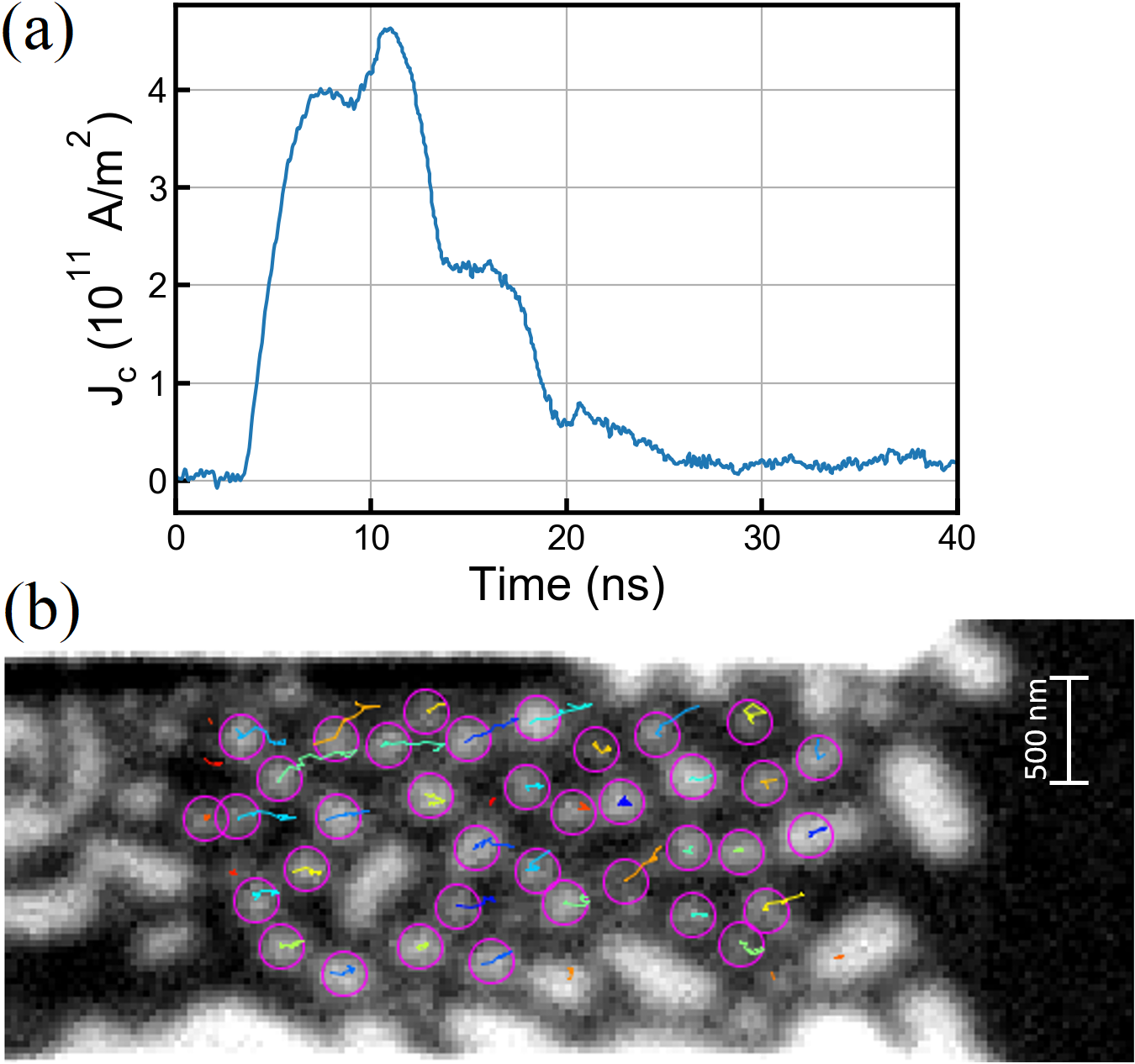


# References

1. [] K. Litzius, I. Lemesh, B. Krüger, P. Bassirian, L. Carette, K. Richter, F. Büttner, K. Sato, O. A. Tretiakov, K. Förster *et al*., “Skyrmion Hall effect revealed by direct time-resolved X-ray microscopy” *Nature Physics* **13**, 170-175 (2017). [↑](#endnote-ref-2)
2. []K. Litzius, J. Leliaert, P. Bassirian, D. Rodrigues, S. Kromin, I. Lemesh, J. Zazvorka, K. Lee, J. Mulkers, N. Kerber *et al*., “The role of temperature and drive current in skyrmion dynamics” *Nature Electronics* **3**, 30-36 (2020). [↑](#endnote-ref-3)
3. []R. Juge, S. G. Je, D. S. Chaves, L. D. Buda-Prejbeanu, J. Peña-Garcia, J. Nath, I. M. Miron, K. G. Rana, L. Aballe, M. Foerster, F. Genuzio, T. O. Mentes, A. Locatelli, F. Maccherozzi, S. S. Dhesi, M. Belmeguenai, Y. Roussigné, S. Auffret, S. Pizzini, G. Gaudin, J. Vogel, and O. Boulle, “Current-Driven Skyrmion Dynamics and Drive-Dependent Skyrmion Hall Effect in an Ultrathin Film”, *Phys. Rev. Appl*. **12**, 044007 (2019). [↑](#endnote-ref-4)
4. []S. Woo, K. Litzius, B. Krüger, M. Im, L. Caretta, K. Richter, M. Mann, A. Krone, R. M. Reeve, M. Weigand *et al*., “Observation of Room-Temperature Magnetic Skyrmions and Their Curent-Driven Dynamics in Ultrathin Metallic Ferromagnets” *Nature Materials* **15**, 501-506 (2016). [↑](#endnote-ref-5)
5. [] J. Bass and W. P. Pratt, “Spin-diffusion lengths in metals and alloys, and spin-flipping at metal/metal interfaces: An experimentalist’s critical review”, *J. Phys.: Condens. Matter* **19**, 183201 (2007). [↑](#endnote-ref-6)
6. [] S. Halas and T. Durakiewicz, “Work functions of elements expressed in terms of the Fermi energy and the density of free electrons”, *J. Phys.: Condens. Matter* **10**, 10815 (1998). [↑](#endnote-ref-7)
7. [] S. Zhang, P. M. Levy, and A. Fert, “Mechanisms of SpinPolarized Current-Driven Magnetization Switching”, *Phys. Rev. Lett.* **88**, 236601 (2002). [↑](#endnote-ref-8)
8. [] C. Petitjean, D. Luc, and X. Waintal, “Unified Drift-Diffusion Theory for Transverse Spin Currents in Spin Valves, Domain Walls, and Other Textured Magnets”, *Phys. Rev. Lett.* **109**, 117204 (2012). [↑](#endnote-ref-9)
9. [] W. Zhang, W. Han, X. Jiang, S. H. Yang, and S. S. P. Parkin, “Role of transparency of platinum–ferromagnet interfaces in determining the intrinsic magnitude of the spin Hall effect” *Nature Physics* **11**, 496 (2015). [↑](#endnote-ref-10)
10. [] C. R. MacKinnon, S. Lepadatu, T. Mercer and P. R. Bissell, “Role of an additional interfacial spin-transfer torque for current-driven skyrmion dynamics in chiral magnetic layers”, *Phys. Rev. B* **102**, 214408 (2020). [↑](#endnote-ref-11)
11. [] G. K. Reeves and M. W. Lawn, “Resistivity measurements of thin film iridium on silicon”, *Journal of Vacuum Science & Technology A* **10**, 3203 (1992). [↑](#endnote-ref-12)
12. [] W. Zhang, M. B. Jungfleisch, W. Jiang, J. Sklenar, F. Y. Fradin, J. E. Pearson, J. B. Ketterson, and A. Hoffmann, “Spin pumping and inverse spin Hall effects—Insights for future spin-orbitronics”, *J. Appl. Phys.* **117**, 172610 (2015). [↑](#endnote-ref-13)
13. [] M. Tokaç, S. A. Bunyaev, G. N. Kakazei, D. S. Schmool, D. Atkinson, and A. T. Hindmarch, “Interfacial Structure Dependent Spin Mixing Conductance in Cobalt Thin Films”, *Phys. Rev. Lett.* **115**, 056601 (2015). [↑](#endnote-ref-14)
14. [] S. Lepadatu, “Effect of inter-layer spin diffusion on skyrmion motion in magnetic multilayers” *Scientific Reports* **9**, 9592 (2019). [↑](#endnote-ref-15)
15. [] W. Legrand, J.-Y. Chauleau, D. Maccariello, N. Reyren, S. Collin, K. Bouzehouane, N. Jaouen, V. Cros, A. Fert, “Hybrid chiral domain walls and skyrmions in magnetic multilayers” *Science Advances* **4**, 0415 (2018). [↑](#endnote-ref-16)
16. [] S. Lepadatu, “Efficient computation of demagnetizing fields for magnetic multilayers using multilayered convolution” *J. Appl. Phys.* **126**, 103903 (2019). [↑](#endnote-ref-17)
